# Benefits of using magnesium sulphate (MgSO_4_) for eclampsia management and maternal mortality reduction: lessons from Kano State in Northern Nigeria

**DOI:** 10.1186/1756-0500-5-421

**Published:** 2012-08-08

**Authors:** Ekechi Okereke, Babatunde Ahonsi, Jamilu Tukur, Salisu Mohammed Ishaku, Ayodeji Babatunde Oginni

**Affiliations:** 1 Population Council, Abuja, Nigeria; 2 Department of Obstetrics and Gynaecology, Bayero University/Aminu Kano Teaching Hospital, Kano, Nigeria

**Keywords:** Magnesium sulphate, Maternal mortality, Millennium Development goals, Northern Nigeria

## Abstract

**Background:**

Despite clear emphasis through the Millennium Development Goals, the problem of high maternal mortality persists especially within low and middle income countries. Various studies report remarkably high maternal mortality rates in northern Nigeria, where maternal mortality rates exceed 1,000 deaths per 100,000 live births and eclampsia contributes approximately 40% of maternal deaths. Across Nigeria, diazepam is routinely used for the management of eclampsia. Prior to February 2008, diazepam was widely used for the management of eclampsia in Kano State (within northern Nigeria) with case fatality rate being over 20%. While magnesium sulphate (MgSO_4_) is recognized as the most effective drug for the management of eclampsia; this study aims to compare MgSO_4_ therapy with diazepam therapy in terms of case fatality rates and costs.

**Findings:**

This retrospective study, including 1045 patients with eclampsia and pre-eclampsia during the years 2008 and 2009, reports a drop in case fatality rates from 20.9% (95% CI: 18.7, 23.2) to 2.3% (95% CI: 1.4, 3.2) among eclampsia patients following the MgSO_4_ intervention. The study observed no significant difference in the cost of using MgSO_4_ therapy compared to diazepam therapy.

**Conclusions:**

The study found a remarkable reduction in case fatality rate due to eclampsia in those who received MgSO_4_ therapy with minimal increase in costs when compared to diazepam therapy. Concerted efforts should be focused on properly introducing MgSO_4_ into emergency obstetric protocols especially within developing countries to reduce maternal mortality and also impact on health system performance.

## Findings

### Introduction

Maternal mortality in the developing world is receiving increasing attention in recent years [1-4] and significantly it is a key emphasis of the Millennium Development Goals (MDGs). The World Health Organization (WHO) reports that hemorrhage/bleeding, infections, unsafe abortions and eclampsia are common causes of maternal mortality especially in developing countries [5]. Severe pre-eclampsia and eclampsia-related deaths are common causes of *preventable* maternal deaths with 99% of these deaths occurring in low and middle income countries [6,7].

Eclampsia is a major cause of maternal morbidity and mortality in Nigeria. It was reported to have contributed to 46.3% of maternal deaths in Kano State [8] and 43% of maternal deaths in Jigawa State [9]. These figures indicate remarkably high rates of maternal mortality with high contributions from eclampsia-related maternal deaths in northern Nigeria.

WHO has recommended the use of magnesium sulphate (MgSO_4_) as a safe and low-cost drug to manage severe pre-eclampsia and eclampsia cases [10]. Studies have shown that the drug significantly lowers the possibility of seizures in women with severe pre-eclampsia or eclampsia, prevents progression from severe pre-eclampsia to eclampsia and generally lowers maternal mortality [11-14]. In many high income countries, magnesium sulphate has been the drug of choice for over 20 years, but in low and middle income countries diazepam, phenytoin and lytic cocktail are still widely used [6,15]. The limited use of magnesium sulphate in many developing countries is attributable to a number of factors such as lack of or poor dissemination of guidelines on the use of the drug, lack of training for health workers on how to administer the drug and the perceived need for monitoring of patients on the drug in intensive care units. Furthermore because the drug is not very expensive, there is limited incentive for drug manufacturers to produce the drug on a commercial scale [16] as well as the fact that prepackaged doses of less effective drugs are more readily available [6].

The objective of the study was to compare MgSO_4_ therapy with diazepam therapy among eclampsia patients across ten secondary health facilities in Kano State within northern Nigeria especially with respect to impact on case fatality rates and cost implications.

### Methods

#### Study design and study sites

This retrospective study was based on an intervention carried out in Kano State within north-western Nigeria, which has 44 local government areas (LGAs) and an estimated total population of 9,401,288 according to the 2006 Nigerian national census [17]. Kano State has a demographic and reproductive health profile that includes a maternal mortality ratio of over 1,000 deaths per 100,000 live births, a relatively high total fertility rate of over 7 births per woman, with 45% of adolescents aged 15 – 19 years having already begun childbearing. Furthermore the state like much of north-western Nigeria is characterized by a low age at first marriage for women (less than 17 years), infant mortality rate of over 110 deaths per 1,000 live births and a modern contraceptive prevalence rate of less than 5% [18,19]. The selected sites for the MgSO_4_ intervention were ten secondary level health facilities serving over half the total population of Kano State and spread across the State; these health facilities are managed and maintained by the Kano state government through the State Health Management Board. Although MgSO_4_ has been approved for marketing and usage in Nigeria, it has not been routinely used in health facilities for eclamptic cases.

Health workers (doctors and midwives) from the ten health facilities were trained on how to use MgSO_4_ through a two day training workshop in January 2008. A simple protocol for the use of the drug was developed and then utilized during the training. The participants were taught how to detect MgSO_4_ toxicity using clinical methods and how to administer the antidote (calcium gluconate). After the training, MgSO_4_was supplied by the state government with support from Population Council to the health facilities to replace diazepam as the drug of choice for the management of severe pre-eclampsia and eclampsia. The training of the health workers ensured that all patients with eclampsia across the ten health facilities received similar treatment with MgSO_4_. In addition, regular supportive supervisory visits were conducted to all the ten facilities to ensure adherence to protocol. Ethical clearance for the intervention was granted by the Kano State Health Management Board as well as Population Council’s Ethical Research Review Committee.

#### Study sample

The study sample consists of ^a^1233 eclampsia patients from the baseline period (2006 – 2007) who received diazepam for the management of eclampsia and 996 eclampsia patients from the intervention period who received MgSO_4_ after presenting to the ten secondary level health facilities selected for the intervention. Similarly there were 49 severe pre-eclampsia patients during the intervention period who also received MgSO_4_. This makes a total of 1045 (severe pre-eclampsia and eclampsia) patients for the intervention period i.e. between February 2008 and January 2009.

The case fatality rate of patients who received diazepam during the baseline study period was estimated by analyzing data on case fatality for eclampsia from representative facilities across the state. Similarly the case fatality rate was estimated for patients who received MgSO_4_ in the ten selected sites during the intervention period. The case fatality rate for eclampsia is defined as the number of deaths with eclampsia as the underlying cause of death per 100 cases of eclampsia that received MgSO_4_ in the general hospitals. The relative standard error (RSE) was used as a measure of an estimated CFR’s reliability; it was obtained by dividing the standard error of the estimate (SE) by the estimate itself (CFR)*100. RSE < 30% was considered reliable.

#### Data collection and analysis

Data for the study was obtained from the obstetric records of eclamptic patients. Data collection did not involve collecting data directly from individual patients; however data was collected from the health records of eclamptic patients with no unique identifiers. Data analysis was done using SPSS statistical software version 15. Comparison was made between diazepam and MgSO_4_ therapy particularly in terms of case fatality rates and also cost implications.

#### ^b^Costs estimation

The estimated cost of using diazepam versus MgSO_4_ for managing eclampsia cases was calculated by applying the cost per ampoule for both diazepam and MgSO_4_ to the expected number of ampoules required for managing patients. The cost of managing recurrent convulsions after a loading dose of MgSO_4_ during the intervention period was estimated. The extra cost of replacing diazepam with MgSO_4_ as the drug of choice for managing eclampsia per eclampsia patient was also calculated.

### Results

Table 1 shows the key characteristics and demographic profile of the intervention group. Fifty two percent (52%) of the patients were teenagers and thirty two percent (32%) were young women. Sixty percent (60.4%) were primigravidae and about four percent (3.7%) were grand multiparous. Very few women had primary education and about seventy four percent (74.1%) were illiterate. Seventy one percent (71%) were in monogamous relationships and about twenty nine percent (28.8%) had polygamous relationships. Table 2 shows the case fatality rates among eclampsia patients at baseline as well as during the intervention period. There was a drastic reduction in case fatality rates among eclampsia patients from 20.9% (95% CI: 18.7, 23.2) to 2.3% (1.4, 3.2) when baseline mortality rates were compared to the intervention period using MgSO_4_ therapy. Table 3 highlights the number and percentage of patients with recurrent convulsions among the eclampsia patients after a loading dose of MgSO_4_ was administered during the intervention period. It shows that more than 90 percent of patients (92.5%) treated with MgSO_4_ did not have recurrent fits. When a recurrent convulsion occurred, it was treated using 2grammes of MgSO_4_ (almost an ampoule of MgSO_4_) administered intravenously. Also about two percent (2%) of the patients who had clinical toxicity were detected by loss of deep tendon reflex using a patellar hammer. All cases of clinical toxicity were treated by stopping MgSO_4_ and further treatment with 1gramme of 10% calcium gluconate. Table 4 shows the estimated costs for managing eclampsia cases with MgSO_4_ compared with diazepam as well as the cost for treating recurrent convulsions using MgSO_4_. Expert advice from clinicians working within the state recommends using about 5 ampoules of diazepam/per day for 2 days which is estimated at N1000 (~6.67 US dollars) to manage a patient with eclampsia. The Pritchard regimen with intramuscular maintenance doses has been widely recommended for use in low resource settings [20]. A modified version of the Pritchard regimen i.e. using about 8 ampoules of MgSO_4_ within a 24 hour period, estimated at N1600 (~10.6 US dollars), was administered to patients in the health facilities during the intervention period. These figures are calculated based on the assumption that diazepam costs N100 per ampoule while MgSO_4_ costs N200 per ampoule, but these costs vary widely across Nigeria. In addition, since just about an ampoule will be required to treat recurrent convulsions, the cost for treating any recurrent convulsions was estimated at N200 (~1.33 US dollars). 

**Table 1:** **Key characteristics and demographic profile of the patients that received MgSO**_**4**_

**Basic characteristics**	**Pre-eclampsia patients (n = 49)**	**Eclampsia patients (n = 996)**	**Total (n = 1045)**
	**N (%)**	**N (%)**	**N (%)**
**Age (years)**			
15-19	19(38.8)	519(52.1)	538(51.5)
20-24	19(38.8)	312(31.3)	331(31.7)
25-48	10(20.4)	148(14.9)	158(15.1)
Unknown	1(2.0)	17(1.7)	18(1.7)
**Parity**			
0	30(61.2)	601(60.3)	631(60.4)
1-5	13(26.5)	351(35.2)	364(34.8)
>5	6(12.2)	33(3.3)	39(3.7)
Unknown	0	11(1.1)	11(1.1)
**Educational status**			
None	36(73.5)	738(74.1)	774(74.1)
Nursery	3(6.1)	39(3.9)	42(4.0)
Primary	4(8.2)	138(13.9)	142(13.6)
Secondary/Vocational	4(8.2)	59(5.9)	63(6.0)
Tertiary	1(2.0)	4(0.4)	5(0.5)
Unknown	1(2.0)	18(1.8)	19(1.8)
**Marital status**			
Married (monogamous)	37(75.5)	705(70.8)	742(71.0)
Married (polygamous)	12(24.5)	289(29.0)	301(28.8)
Unknown	0	2(0.2)	2(0.2)
**Blood pressure on admission**			
Normal (SBP ≤ 139/DBP ≤ 89)	0	42 (4.2)	42 (4.2)
Hypertensive (SBP ≥ 140/DBP ≥ 90)	48 (98.0)	940 (94.4)	988 (94.5)
Unknown	1 (2.0)	14 (1.4)	15 (1.4)

**Table 2:** Eclampsia case-fatality rates at baseline and during the intervention period

	**All cases (n)**	**Fatality (n)**	**CFR(95%CI) (%)**	**RSE (%)**
Pre-intervention period				
(Eclampsia patients)	1233	258	20.9(18.7, 23.2)	5.5
Intervention period				
(Eclampsia patients)	996	23	2.3(1.4, 3.2)	20.9

*CFR*= Case-Fatality Rate. *RSE*= Relative standard error.

**Table 3:** **Recurrent convulsions among the patients with eclampsia after MgSO**_**4**_**loading dose**

**Had Recurrent convulsions after the loading dose**	**N (%)**
Yes	62 (6.2)
No	921 (92.5)
Unknown	13 (1.3)
Total	996 (100)

**Table 4:** **Estimated cost per patient of using MgSO**_**4**_** and Diazepam for eclampsia management**

	**Unit cost/ampoule**	**Ampoules required**	**Total cost/episode**
Diazepam	N 100	10	N 1000 ($6.67)*
MgSO_4_ (loading dose)	N 200	8	N 1600 ($10.6)*
MgSO_4_ (Recurrent convulsions)	N 200	1	N 200 ($1.33)*

### Discussion

This study illustrates a reduction in case fatality rates and the benefits of using MgSO_4_ for the management of eclampsia. This finding reiterates the results of the Magpie trials where MgSO_4_ reduced the relative risk of eclampsia by half without significant harmful effects on the mother [12] as well as a review of seven randomized controlled trials involving 1396 women in which MgSO_4_ reduced maternal deaths with a risk ratio of 0.59 (95% CI 0.38 to 0.92) [21]. The results from this study also reinforce the strong recommendation from the WHO on the use of MgSO_4_ over other anticonvulsants for the treatment of severe preeclampsia and eclampsia [22].

At present the estimated cost of managing a convulsion and preventing further episodes of eclampsia using MgSO_4_ is slightly over 10 US dollars in Nigeria compared with about 7 US dollars if diazepam is utilized. As illustrated by this study less than 1 in 10 patients given a loading dose of MgSO_4_ will likely get recurrent convulsions. The extra cost per person per episode for managing eclampsia that will be incurred if MgSO_4_ replaces diazepam is estimated to be between 3 to 4 US dollars. For MgSO_4_ to be available at cheaper prices, two key strategies are recommended. First, there is need for the Nigerian government to ensure proper price control mechanisms for the drug are put in place while offering market incentives to encourage local production of the drug. Second, there maybe need to introduce subsidies for MgSO_4_ as presently occurs with anti-retroviral drugs [23].

It is expected over time that the cost of introducing MgSO_4_ into health facilities and sustaining its use within the wider health system should progressively reduce, especially if a well organized and efficient supply chain management system for the drug is put in place. Furthermore costs for the procurement of MgSO_4,_ for the management of eclampsia, when applied at national levels, should certainly be manageable, if proper economies of scale are factored into the procurement of the drug.

The significantly better clinical outcomes highlighted through the MgSO_4_ intervention provides firm justification for the need to strongly promote the routine use of MgSO_4_ in health facilities across Nigeria and not just in Kano State. There will be a cumulative impact on the per capita income and gross domestic product (GDP) in Kano State and across the country if this relatively low-cost medication is effectively utilized in the fight against maternal mortality.

With clear clinical and economic benefits associated with using MgSO_4_ for eclampsia management, governments in Nigeria and other low income countries should work towards producing clear national guidelines on the use of MgSO_4_ in concurrence with WHO guidelines. Ministries of Health at state and national levels could also create more awareness about the benefits of using the drug, in collaboration with the World Health Organization as indicated in a previous publication [23]. Appropriate trainings for health care workers on the use of the drug should be a priority. Furthermore there should be necessary policy changes to ensure that the drug is included in the essential drug list at both state and national levels.

Following the acknowledgement of the importance of using MgSO_4_ for managing eclampsia by WHO, UNFPA, UNICEF and the World Bank [24] and the successes of the MgSO_4_ intervention in Kano State by Population Council, the Kano State government has taken over the supply of the drug to its health facilities. Preliminary results indicate clear benefits which should justify the continued use of MgSO_4_ in the free maternal and child programme currently run by the Kano State government. But a key challenge will be to ensure that there is a steady provision of drugs and supplies to health facilities as well as for patients in need. Freedman and others proposed [25] that governments in tackling the issue of high maternal mortality, should address the problem from a health systems perspective such that possible catastrophic costs and poor health services especially experienced by the poorest populations are equally addressed. In the final analysis, the use of MgSO_4_ for eclampsia management may not entirely be a “magic bullet” but it should be properly integrated into the implementation framework for maternal mortality reduction within the health systems of resource limited countries, for there to be any significant impact on health system performance. As suggested by Nyamtema and others [4], policy makers should ensure that such key lessons are translated into concrete action plans for achieving the goals set for maternal and child health in order to achieve MDGs 4 and 5 across the developing world.

In conclusion, MgSO_4_ therapy reduced the case fatality rate due to eclampsia to a significantly greater extent than the existing diazepam therapy in ten intervention centres in Kano State. There is no significant difference in costs in using both drugs. MgSO_4_ therapy can be administered by trained health workers who can recognize the side-effects and the necessity for stopping the drug when needed. The continuous supply of the drug by the government is necessary to replace the present day less standard diazepam therapy for eclampsia. Prevention of teenage pregnancy and raising the educational status of women in this region will also help in the long run to reduce maternal mortality due to eclampsia.

### Endnotes

^a^All the 1233 patients who reported during the baseline/pre-intervention period were recorded and treated as eclampsia patients and received diazepam.

^b^To introduce MgSO_4_ into the ten selected health facilities during the intervention period, Population Council incurred expenses that included the costs of training health workers, costs associated with monitoring and evaluation of the MgSO_4_ intervention as well as cost of procurement of the drug for use in the selected health facilities. However the breakdown of these costs was not included in the costs estimation.

*I US Dollar = 150 Naira.

## Competing interests

The authors declare that they have no competing interests.

## Authors’ contributions

EO and SMI jointly participated in the design of the study and the outline for the manuscript. EO and ABO carried out the data collection, data analysis and interpretation of the data. EO developed the first draft of the manuscript. BA, JT and SMI made significant efforts in critically revising the draft for substantial intellectual content. All authors read and approved the final manuscript.
